# Detection of SARS-CoV-2 IgG antibodies and inflammatory cytokines in saliva-a pilot study

**DOI:** 10.1016/j.jobcr.2023.02.008

**Published:** 2023-02-17

**Authors:** S. Wadhwa, A.J. Yoon, K. Kister, I. Bolin, N. Chintalapudi, A. Besmer, A. Cantos, J. Shah, S.K. Gaitonde, S.W. Granger, C. Bryce, R. Fischer, S.B. Eisig, M.T. Yin

**Affiliations:** a Division of Orthodontics, Columbia University Irving Medical Center, New York, NY, USA; b Division of Oral & Maxillofacial Pathology and Department of Pathology & Cell Biology, Columbia University Irving Medical Center, New York, NY, USA; c Department of Medicine Infectious Disease, Columbia University Irving Medical Center, New York, NY, USA; d Salimetrics, LLC, Carlsbad, CA, USA; e Division of Oral & Maxillofacial Surgery, Columbia University Irving Medical Center, New York, NY, USA

**Keywords:** COVID-19, Saliva, Coronavirus, Inflammation, Oral-systemic disease(s), Xerostomia

## Abstract

**Objective:**

The pandemic caused by SARS-CoV-2 virus continues to have a profound effect worldwide. However, COVID-19 induced oral facial manifestations have not been fully described. We conducted a prospective study to demonstrate feasibility of anti–SARS-CoV-2 IgG and inflammatory cytokine detection in saliva. Our primary objective was to determine whether COVID-19 PCR positive patients with xerostomia or loss of taste had altered serum or saliva cytokine levels compared to COVID-19 PCR positive patients without those oral symptoms. Our secondary objective was to determine the correlation between serum and saliva COVID-19 antibody levels.

**Materials and methods:**

For cytokine analysis, saliva and serum were obtained from 17 participants with PCR-confirmed COVID-19 infection at three sequential time points, yielding 48 saliva samples and 19 paired saliva-serum samples from 14 of the 17 patients. For COVID-19 antibody analyses, an additional 27 paired saliva-serum samples from 22 patients were purchased.

**Results:**

The saliva antibody assay had 88.64% sensitivity [95% Confidence Interval (CI) 75.44%, 96.21%] to detect SARS-CoV-2 IgG antibodies compared to serum antibody. Among the inflammatory cytokines assessed - IL-6, TNF-α, IFN-γ, IL-10, IL-12p70, IL-1β, IL-8, IL-13, IL-2, IL-5, IL-7 and IL-17A, xerostomia correlated with lower levels of saliva IL-2 and TNF-α, and elevated levels of serum IL-12p70 and IL-10 (p < 0.05). Loss of taste was observed in patients with elevated serum IL-8 (p < 0.05).

**Conclusions:**

Further studies are needed to construct a robust saliva-based COVID-19 assay to assess antibody and inflammatory cytokine response, which has potential utility as a non-invasive monitoring modality during COVID-19 convalescence.

## 1 Introduction

The coronavirus disease 2019 (COVID-19) has been shown to be transmitted through both aerosol transmission (cough, sneeze, droplet inhalation) and contact methods (contact with oral, nasal, ocular mucous membranes).^1^ The oral cavity is one of the first entry points between the external environment and the body, a pathway that plays a critical role at the onset of COVID-19. SARS-CoV-2 enters the cells through the human angiotensin-converting enzyme 2 (ACE2) receptor. The ACE2 positive cells are abundantly found in the oral cavity including the salivary duct epithelium, tongue, buccal mucosa and gingiva.^2^ Autopsy studies confirmed *in vivo* infection of the salivary glands and oral mucosa.^3^ In the first ten days, SARS-CoV-2 accumulated in the oral mucosa and persisted up to 18 days on average in asymptomatic individuals with an increased oral viral load being associated with the severity of the COVID-19 symptoms.^3^^,^^4^ Recent studies of subjects infected with SARS-CoV-2 found oral manifestations in 45% of cases with loss of taste and dry mouth being the most common.^5^, ^6^, ^7^ Further, loss of taste has been shown to been present in greater than 50% of people who get infected with COVID-19 after complete vaccination.^8^

Saliva is composed of salivary gland secretions and gingival crevicular fluid. Saliva has been shown to be a sensitive source of detection for SARS-CoV-2.^9^, ^10^, ^11^ The viral RNA, that can be detected in saliva of many infected patients, peaks 7–10 days after symptom onset and persist ≥20 days after symptom onset in one third of the cases.^12^ However, the literature is inconclusive regarding the test performance using saliva vs. upper respiratory tract excretions for SARS-CoV-2.^13^, ^14^, ^15^

The detection of the anti-SARS-CoV-2-specific antibodies in saliva has been documented.^16^ However, less is known how SARS CoV-2 antibody titers in the saliva correlate with those measured in the serum.^17^, ^18^, ^19^ The SARS-CoV-2 infection is associated with marked increase in serum cytokines that promote tissue damage and inflammation (cytokine storm) in severely ill patients^20^; however, the response of salivary and serum cytokines to SARS- CoV-2 infection induced oral manifestations has not yet been studied in depth.^21^ Our primary objective was to determine whether COVID-19 PCR positive patients with xerostomia or loss of taste had altered serum or specific saliva cytokine levels compared to COVID-19 positive patients without those oral symptoms. Our secondary objective was to determine the correlation between serum and saliva COVID-19 antibody levels, in order to determine whether saliva alone can be used for COVID-19 antibody monitoring. To achieve our objectives, we assessed anti-SARS-CoV-2-Nucleocapsid-specific antibodies and inflammatory cytokines in saliva and serum samples prospectively collected from COVID-19 positive patients with and without oral symptoms.

## 2 Materials and Methods

### 2.1 Subjects

The study conforms to STROBE Guidelines. Participants were recruited from the Columbia University Irving Medical Center (CUIMC) observational prospective cohort study ‘Determinants of SARS-CoV-2 persistence after convalescence’ (IRB AAAS9722, approval date: 3/26/2020). Candidates were eligible to participate if they had received a PCR laboratory confirmed diagnosis of COVID-19, and were at least 7 days post onset of COVID-19 symptoms (fever, cough, shortness of breath). Participants were consecutively selected from May 2020 to August 2020, when we stopped due to the lack of new COVID-19 PCR positive patients enrolling in the study for greater than 2 weeks.

Participants were followed for 3 weeks (baseline-time of enrollment, 1 week, and 2 weeks post enrollment). Two study participants missed one or two study visits. At baseline, the participants filled out a survey that included demographic information and details of symptoms experienced. Saliva samples for this study were collected at each visit. Approximately 1–1.8 ml of saliva samples were collected by asking participants to passively drool into the saliva collecting tube (Salimetrics LLC, Carlsbad, Ca). Immediately after collection, saliva samples were kept in −80 °C freezer. For another study, serum samples were also collected for each visit. However, for this study we were only able to obtain one serum sample from 14 of the enrolled patients at baseline and additional sample 1 week post baseline for 5 of the enrolled subjects. Therefore, for cytokine testing we had 48 saliva samples from 17 patients and 19 paired serum samples from 14 patients.

For antibody testing, an additional 27 SARS-CoV-2 positive serum and saliva samples were purchased from Cantor BioConnect from 22 patients; these samples were collected from various sites across the US, approximately 2 weeks after the PCR diagnosis of COVID-19. Therefore, for serum and saliva antibody testing we had 46 paired serum-saliva samples from 36 patients (Table 1).

**Table 1:** Participant and specimen characteristics.

Participant Characteristics			
	CUIMC (n = 17)	Cantor Bioconnect (n = 22)	Total (n = 39)
Age			
Median (Interquartile range)	43 (19)	38 (13)	39 (15)

Gender			
Male	8	8	16
Female	9	14	23
Race			
White/Non-Hispanic	9	n/a	9
Black/Non-Hispanic	1	n/a	1
Hispanic	4	n/a	4
Asian	3	n/a	3
Pre-existing medical conditions			
Chronic Heart Disease			
Chronic Obstructive pulmonary disease	1	n/a	1
Hypertension			
Diabetes Mellitus	3	n/a	3
HIV			
Cancer	1	n/a	1
None of the above	1	n/a	1
	1	n/a	1
	1	n/a	1
	12	n/a	12
Hospitalization	3	n/a	3
Loss of taste at baseline			
Yes	13	n/a	13
No	4	n/a	4
Xerostomia at baseline			
Yes	10	n/a	10
No	5	n/a	5
Not Reported	2	n/a	2

Specimen Characteristics			
Saliva	48 specimens	27 specimens	75 saliva specimens
	(from 17 participants)	(from 22 participants)	(from 39 participants)
Serum	19 specimens	27specimens	46 specimens
	(from 17 participants)	(from 22 participants)	(from 36 participants)

In sum, a total of 75 saliva samples and 46 paired serum samples collected at 1–3 sequential time points from 39 COVID-19 positive participants were available for analyses.

### 2.2 Serologic and saliva assays of anti-SARS-CoV-2 antibodies

Anti-SARS-CoV-2 N-protein specific IgG antibodies in serum and saliva samples were detected using ELISA assays at a commercial laboratory [KT-1032 kit for serum (Epitope Diagnostics, Inc.), 1–1260 kit for saliva, Salimetrics LLC]. Briefly, antibodies in test samples were detected using an anti-human IgG detection antibody linked to horseradish peroxidase (HRP). After each incubation, unbound components were washed. Anti-Human IgG HRP enzyme conjugates were added and the levels measured by the reaction of the HRP enzyme to the substrate tetramethylbenzidine (TMB). The optical density (OD) was read on a standard plate reader at 450 nm with a 630 nm subtraction. The total amount of anti-IgG HRP enzyme conjugate detected was proportional to the amount of anti-SARS CoV-2 IgG present in the sample. In addition, total IgG levels were quantified using the Salimetrics total IgG assay kit following manufacturer's protocol (1–4502 kit, Salimetrics Inc.). Two Samples that had total IgG of less than 3 μg/ml were excluded from analysis. The OD value obtained from the N-protein specific kit was normalized against the total IgG levels, and samples above the recommended cut-off of the resulting normalized value were considered positive for the presence of N-protein specific antibodies. Antibody for the N-protein was performed on 44 saliva-serum pairs from 34 patients.

### 2.3 Inflammatory cytokine assays in saliva

The inflammatory cytokine assays were performed in 19 serum samples paired with 48 saliva samples collected at different time points for those with available COVID-19 symptoms information (total n = 67). The pro-inflammatory cytokines IL-6, TNF-α, IFN-γ, IL-10, IL-12p70, IL-1β, IL-8, IL-13, IL-2, IL-7, IL-17A, IL-5 and IL-7 were assessed using the commercial chemiluminescent ELISA kit (Meso Scale Diagnostics, Rockville, MD) according to manufacturer's instructions for the serum. For the saliva, samples were run following manufacturer's instructions using a minimum required sample dilution specific for saliva and adjustments to the standards to accommodate lower analyte levels in saliva and so measured levels were interpolated from the calibration curve.

### 2.4 Statistical analysis

Spearman correlations were performed to assess association between saliva and serum antibody levels. One-way analysis of variances (ANOVA) was used to assess correlation between the inflammatory cytokine levels and the symptoms (dry mouth, loss of taste) in both saliva and serum. Statistical analyses were conducted using IBM SPSS Statistics version 25 and p < 0.05 was considered as statistically significant.

## 3 Results

### 3.1 Subjects

Between May and August of 2020, seventeen consecutive PCR positive COVID-19 patients were enrolled in the CUIMC prospective cohort, baseline saliva and blood samples were obtained at an average of 5.7 weeks (SD: 2.7) weeks from the onset of symptoms. Two patients missed one or two scheduled study visits. In the Cantor BioConnect cohort, saliva and serum sample collection occurred at approximately 2 weeks from the time of COVID-19 diagnosis. Overall, the age of participants varied from 10 to 61 years of age (Median 39) as shown in Table 1. In the CUIMC cohort (n = 17), one had HIV and three required hospitalizations. All patients recovered from COVID-19 infection without further complication.

Saliva and serum SARS-CoV-2 antigen-specific IgG antibody.

Correlation between saliva and serum SARS-CoV-2 antigen-specific IgG antibodies were assessed in the paired 44 saliva and serum samples. 100% (44 out of 44) had positive serum IgG, and 88.6% (39 out of 44) had positive saliva IgG (Table 2). The saliva antibody assay had 88.64% sensitivity [95% Confidence Interval (CI) 75.44%, 96.21%] to detect SARS-CoV-2 IgG antibodies, compared to the serum assay.

**Table 2:** SARS-CoV-2 antigen-specific IgG detection in 44 paired serum and saliva specimens.

N-Protein	Serum	Saliva
Positive	44 (100%)	39 (88.63%)
Negative	0	5 (11.37%)

## 4 Inflammatory cytokines in saliva and serum

Expression levels of IL-6, TNF-α, IFN-γ, IL-10, IL-12p70, IL-1β, IL-8, IL-13, IL-2, IL-7, IL-17A, IL-5 and IL-7 were measured in saliva and serum pairs obtained from the COVID-19 patients with available baseline symptoms information. Elevated levels of all but one (IL-12 p70) inflammatory cytokines were detected in saliva compared to serum and saliva cytokine levels had more variance than the serum cytokines (Table 3). Based on the one-way analysis of variance (ANOVA), the individuals who experienced dry mouth had significantly lower levels of saliva IL-2 and TNF-α, and higher levels of serum IL-12p70 and IL-10 (Table 4). In patients experiencing loss of taste, higher level of serum IL-8 was detected (p = 0.048).

**Table 3:** Inflammatory cytokine levels in COVID-19 positive patients’ saliva and serum.

	IFN-γ	IL-10	IL-12p70	IL-13	IL-1β	IL-2	IL-6	IL-8	TNF-α	IL-17A	IL-5	IL-7
Saliva												
Mean (SD) (pg/mL)	5.58 (12.57)	0.76 (0.45)	0.51 (0.30)	6.69 (3.32)	108.49 (117.82)	0.80 (0.57)	4.38 (4.44)	687.64 (650.03)	3.16 (2.39)	2.27 (11.61)	0.61 (3.11)	15.05 (8.10)
Min-Max (pg/mL)	0.94–80.29	0.18–2.16	0.15–1.74	2.05–16.9	6.55–544.21	0.11–2.59	1.03–25.21	141.74–3373.5	0.4–9.99	0.06–80.08	0.02–21.59	1.66–31.35
Range	80.29	1.98	1.59	14.85	537.66	2.48	24.18	3231.76	9.95	80.02	21.57	29.69
Serum												
Mean (SD) (pg/mL)	4.37 (3.46)	0.42 (0.21)	0.54 (0.23)	4.52 (1.67)	0.26 (0.37)	0.71 (0.31)	1.27 (0.79)	5.09 (4.64)	1.01 (0.37)	0.89 (0.88)	0.42 (0.24)	6.36 (6.98)
Min-Max (pg/mL)	1.92–17.24	0.09–0.85	0.2–1	0.7–7.27	0.01–1.32	0.08–1.24	0.47–3.48	2.04–23.51	0.25–1.9	0.24–4.04	0.07–0.93	0.05–32.44
Range	15.32	0.76	0.8	6.57	1.31	1.16	3.11	21.47	1.65	3.8	0.86	32.39

Fold Increase in Saliva	1.27	1.80	0.93	1.48	408.3	1.12	3.43	135.15	3.12	2.54	1.47	2.37

**Table 4:** Mean value and standard deviation of the inflammatory cytokine levels in saliva and serum, comparing those with or without xerostomia and loss of taste at baseline visit. ANOVA *p < 0.05

Cytokines	Sample Matrix	Dry Mouth		p-value
		Yes	No	
IL-2 (pg/mL)	Saliva	0.518 (0.295)	0.938 (0.518)	0.018
TNF-α (pg/mL)	Saliva	1.854 (1.040)	3.939 (2.642)	0.006
IL-12p70 (pg/mL)	Serum	0.718 (0.224)	0.470 (0.224)	0.022
IL-10 (pg/mL)	Serum	0.562 (0.227)	0.358 (0.167)	0.041
		Loss of Taste		
		Yes	No	
IL-8 (pg/mL)	Serum	5.088 (4.636)	4.017 (1.368)	0.048

Very few participants in this study sample were hospitalized (n = 3). Hospitalized participants had higher levels of IL-13 in saliva and serum, higher levels of IL-8 in serum, and lower levels of IL-5 in serum compared to participants who were not hospitalized (p < 0.05 for all).

## 5 Discussion

We found that saliva IL-2 and TNF-α levels were lower, and serum IL-12p70 and IL-10 (p < 0.05) higher in patients with xerostomia than those without. Xerostomia is one of prevalent and persistent oral symptoms associated with COVID-19.^22^ It has been shown that the salivary glands contain the SARS-CoV-2 receptor entry molecules ACE2 and TMPRSS2^23^ and SARS-CoV-2 virus has been found in autopsied saliva gland specimens.^3^ It is speculated that after the cellular entry, SARS-CoV-2 virus could possibly damage saliva glands, causing xerostomia in the early phase of COVID-19.^24^ Consistent with this, we found higher serum levels of Il-10 in COVID-19 patients with xerostomia. Patients with increased salivary disease activity and Sjogren's syndrome have been found to have higher serum levels of IL-10.^25^ The lymphocytes in the salivary glands have been shown to produce IL-2^26^ and IL-2 treatment has been shown to improve salivary secretions in a mouse model of Sjogren's syndrome,^27^ suggesting that Covid-19 could damage the salivary glands causing lower levels of salivary IL-2 and reduced salivary flow. However, it was surprising that TNF-α levels were lower in the saliva in patients with COVID-19 induced xerostomia.^28^ Time course experiments are needed with non-COVID and COVID infected patients to see if saliva inflammatory cytokine levels are initially elevated and then reduced during the progression and convalescence of COVID-19 induced xerostomia.

Loss of taste and smell is a well described symptom of COVID-19.^29^ Higher level of serum IL-8 was detected in COVID-19 patients that experienced loss of taste than in those without (p = 0.048). One study has shown that serum IL-8 levels correlate with COVID-19 clinical symptoms.^30^ Therefore, in our study elevated serum IL-8 levels maybe distinguishing COVID-19 patients with and without clinical symptoms.

Loss of taste and Xerostomia usually recovers 1–3 weeks after COVID-19 infection.^31^^,^^32^ In our study due to the limitation of seeing COVID-19 positive infected patients at the hospital at that time for non-emergency care, the average duration from COVID-19 diagnosis and 1st study visit was 5.7 weeks. Therefore, the patients reporting xerostomia and loss of taste at their baseline visit were on average almost 6 weeks after initial diagnosis and maybe be suffering from longer lasting form of covid induced xerostomia and loss of taste than the Covid −19 typical patients.

In our study, we were able to detect SARS-CoV-2 IgG antibody in both saliva and serum. Positive SARS-CoV-2 antibody was detected as early as 2 weeks following infection. Antibodies were also detected 12–13 weeks after the confirmed infection. Moreover, there was a high correlation between saliva versus serum antibody test, in which saliva assay had 88.64% sensitivity [95% Confidence Interval (CI) 75.44%, 96.21%] to detect SARS-CoV-2 specific antibody compared serum. Our finding is similar to other studies reporting near 100% concordance between saliva and serum antibody positivity in PCR confirmed COVID-19 cases^16^^,^^18^

Our study is limited by a modest sample size, few participants with severe disease, and the first available specimen was obtained approximately 6 weeks after the onset of COVID-19 symptoms. Therefore, we are unable to assess correlations between cytokine levels and COVID-19 PCR cycle threshold values.

## 6 Specific conclusion-

In this paper, we demonstrated feasibility of using saliva for measuring cytokines in COVID-19 induced oral symptoms and anti–SARS-CoV-2 IgG levels. Saliva can be collected non-invasively, which makes saliva-based antibody and cytokine detection an optimal method of COVID-19 surveillance and therapeutics, as previously described.^33^^,^^34^

## Funding

This research did not receive any specific grant from funding agencies in the public, commercial, or not-for-profit sectors.

Footnote for Table 2- The 44 paired specimens were from 34 participants, which included 17 paired specimens (from 12 participants, with adequate total IgG levels) from CUIMC and 27 paired specimens from 22 participants from Cantor BioConnect.

Footnote for Table 3- Cytokine testing was performed on 48 saliva samples from 17 participants and 19 serum samples from 14 participants from CUIMC.

Footnote for Table 4- Cytokine testing was performed on 48 saliva samples from 17 participants and 19 serum samples from 14 participants from CUIMC.
